# Comparing Variants of the Cell-Penetrating Peptide sC18 to Design Peptide-Drug Conjugates

**DOI:** 10.3390/molecules27196656

**Published:** 2022-10-07

**Authors:** Joshua Grabeck, Tamara Lützenburg, Pia Frommelt, Ines Neundorf

**Affiliations:** Department of Chemistry, Institute of Biochemistry, University of Cologne, 50674 Köln, Germany

**Keywords:** peptide-drug conjugates, cell-penetrating peptides, cancer, cytostatic drugs, drug delivery

## Abstract

Herein, the design and synthesis of peptide-drug conjugates (PDCs) including different variants of the cell-penetrating peptide sC18 is presented. We first generated a series of novel sequence mutants of sC18 having either amino acid deletions and/or substitutions, and then tested their biological activity. The effects of histidine substituents were found to be not meaningful for sC18 uptake and cell selectivity. Moreover, building a nearly perfect amphipathic structure within a shortened sC18 derivative provided a peptide that was highly membrane-active, but also too cytotoxic. As a result, the most promising analog was sC18^ΔE^, which stands out due to its higher uptake efficacy compared to parent sC18. In the last set of experiments, we let the peptides react with the cytotoxic drug doxorubicin by Thiol–Michael addition to form novel PDCs. Our results indicate that sC18^ΔE^ could be a more efficient drug carrier than parent sC18 for biomedical applications. However, cellular uptake using endocytosis and resulting entrapment of cargo inside vesicles is still a major critical step to overcome in CPP-containing peptide-drug development.

## 1. Introduction

Cell-penetrating peptides (CPPs) are able to overcome the cell membrane and to shuttle distinct bioactive cargo inside the cell interior [1]. Usually, CPPs comprise a relatively short amino acid (aa) sequence (<30 aa) featuring an amphipathic and/or positively charged character [2,3]. In fact, it has been shown that the positive net charge of CPPs plays an important role in uptake efficiency by supporting the first contact with negatively charged constituents at the outer face of the plasma membrane [4]. Triggered by this electrostatic interaction, CPPs accumulate at the membrane surface and the next steps of the internalization process are initiated. CPPs can be taken up by cells using two main entry mechanisms, namely endocytosis or direct penetration. Endocytosis is an energy-driven pathway and is typically not favored because the CPP-cargo construct ends up in endosomal/lysosomal vesicles. On the other side, direct penetration relies on the transient formation of membrane pores and, thus, may be accompanied by membrane destabilizing effects that are harmful to cells [4]. However, the direct route prevents the entrapment of the CPP-cargo complex inside endosomal vesicles and is, for this reason, the strongly favored mechanism. To achieve efficient endosomal release, various strategies have been explored during the last years, e.g., attaching endosomolytic peptide sequences or small molecules to CPPs, co-incubating CPPs with endosomolytic substances, or designing small and stapled CPPs that are able to follow direct cell entry and circumvent the endosomal pathway [5,6,7,8].

CPPs were discovered more than thirty years ago and during the last decades, a plethora of different CPP sequences has been presented and developed for cargo delivery [9]. The bioactive cargo molecules were combined with the peptide carriers either in a covalent or non-covalent manner. For instance, negatively charged nucleic acids, such as plasmid DNA or various kinds of RNA, are typically complexed with positively charged CPPs, while small (often cytostatic) organic molecules are frequently coupled by chemical means using appropriate linkers. Indeed, the last years have seen an increasing interest in the development of peptide-drug conjugates (PDCs) having CPPs as the peptide part included [10].

In our group, we have designed the CPP named sC18, a 16 aa long peptide, which is derived from the *C*-terminal part of the cationic antimicrobial peptide CAP18 that belongs to the family of cathelicidins [11]. sC18 itself exhibits only very low antimicrobial activity, but we recently demonstrated that sequence modifications result in novel sC18 variants having high antimicrobial activity against different bacteria including pathogenic strains [12]. However, owing to its high membrane-activity, sC18 efficiently interacts with the lipid environment of plasma membranes, too, leading to internalization in various mammalian cell types. Moreover, we have found out that sC18 uses both mechanisms of cell entry, direct penetration, as well as endocytosis, respectively. During the last years, we successfully applied sC18 as a transport system useful in diagnostic and therapeutic cancer research [13,14,15].

Previously, we focused more on structure-relationship studies with the goal of identifying aa residues within the sC18 sequence that are critical for cell selectivity, membrane translocation and efficient drug transport. Following this, we found a shortened sequence that we named sC18*, lacking the last four aa of the *C*-terminus of sC18. Notably, sC18* turned out to be highly effective in bringing bioactive peptide sequences inside the cell interior [16,17]. More recently, we performed an alanine scan and appreciated that replacement of the glutamate in the *C*-terminal region led to much higher cellular uptake compared to the parent sC18 peptide [18]. In addition, when this glutamate was completely deleted, resulting in peptide sC18^ΔE^, promising cellular uptake accompanied by low cytotoxicity was observed [12]. Inspired by those findings, the aim of the current study was to generate and biologically characterize different mutants of both peptides, e.g., sC18* (the shorter one) as well sC18^ΔE^ (lacking the glutamate). Adapted to recent reports in which the positive impact of histidine on cellular uptake and endosomal escape were reported, we designed variants having several residues substituted with histidine [19,20]. One bottleneck in CPP development is lack of specificity and still, no breakthrough for clinical use has been achieved. Thus, novel strategies to tailor CPPs for a targeted delivery would be highly welcome. Since the pKa value of histidine is around 6.0, higher specificity towards the acidic environment of cancer cells was observed for some histidine-rich CPPs [21,22,23]. This effect is related to a pH-dependent increase in total positive net charge leading to higher electrostatic interaction with the negatively charged plasma membrane of cancerous cells. Within this study, we wanted to determine how the beneficial effects of histidine would impact the novel CPP sC18^ΔE^. In a second approach, we were interested in the relevance of the hydrophilic/hydrophobic balance of the shortened sC18 variant sC18*. Despite the importance of positive charges, amphipathicity also plays an important role in membrane interactions and was, therefore, investigated by us [24]. Finally, we assessed the potential of sC18, sC18^ΔE^ and sC18* to deliver doxorubicin in tumor cells.

Our results show that sequence amphipathicity has to be critically adjusted and that the concept of histidine substitution must not necessarily have a positive impact on CPP activity. Furthermore, we highlight that sC18^ΔE^ in conjunction with doxorubicin is a versatile cytostatically acting PDC.

## 2. Results and Discussion

### 2.1. Design and Synthesis of Different Novel sC18 Variants

Within this study, we aimed to generate and biologically characterize different sC18 variants, which were either based on a shorter sC18 peptide lacking the last four amino acids (sC18*) or derived from sC18, in which the glutamate in the *C*-terminal part of its sequence (sC18^ΔE^) was deleted. Therefore, we strategically introduced amino acid substitutions to increase amphipathicity or pH sensitivity.

In the first attempt of optimization, sC18^ΔE^ was taken and the histidine ratio in its sequence was modulated in a way that either all lysine or all arginine residues, respectively, were substituted by histidine, leading to peptides named sC18^ΔE1−2^. sC18^ΔE3−4^ represented variants having a complete part of the helix composed of histidines (see Figure S1).

On the other side, we were eager to improve the activity of sC18*. Although we have recently proven that it is very useful for the cellular import of bioactive peptide sequences, it is itself only fairly taken up by cells [25]. Thus, we designed sC18*^R,L^, a variant bearing a full hydrophobic and hydrophilic face within the helix. The increased amphipathic character was hypothesized to lead to an optimal membrane interaction and cell entry (see Figure S1 for helical wheel projections and Table 1 for peptide sequences). For comparison, we included the peptides sC18, sC18* and sC18^ΔE^ in our studies. All peptides were synthesized via Fmoc/t-Bu solid-phase peptide synthesis and were obtained in high purities (Table 1 and Figures S2–S9). Additionally, a 5(6)-carboxyfluorescein labeled series was generated for later cellular uptake studies (Figures S2–S9).

### 2.2. Analysis of pH-Responsive Histidine Variants

Histidine is known to undergo changes in its protonation state at acidic conditions owing to its pKa value of 6.8. Since the pH value inside endosomes continually decreases during endosome maturation, this effect should lead to a supporting proton-sponge effect of added histidine residues resulting finally in facilitated endosomal escape [26]. In fact, this has been recently observed for several histidine-containing peptides that were used to shuttle bio-responsive siRNA inside cells [27,28]. Moreover, a pH gradient between tumor tissues and the physiological environment exists, and thus, an increased histidine content in CPPs was demonstrated to induce a selective uptake of cancerous cells [29]. To investigate the impact of the introduced histidine residues on the activity of the herein synthesized peptides sC18^ΔE1−4^, they were analyzed at two different pH values, normal physiological pH 7.4 and more acidic pH 6.8 that would be present, e.g., in a tumor environment, or within endosomal vesicles.

We first performed CD spectroscopy measurements at pH 6.8 and pH 7.4, respectively. As shown in Figure S10 all peptides were unstructured in phosphate buffer but formed alpha-helical structures when in the presence of trifluoroethanol (TFE). This observation agreed with already published data of the parent peptide sC18 as well as of sC18^ΔE^ [12,25]. A slight increase in helicity was measured for the R-value (an indicator for helical structure formation) when the peptides encountered a more acidic environment (Table S1). Generally, we concluded that the formed alpha-helical structures would be a helpful prerequisite for efficient membrane interaction.

**Figure 1:**
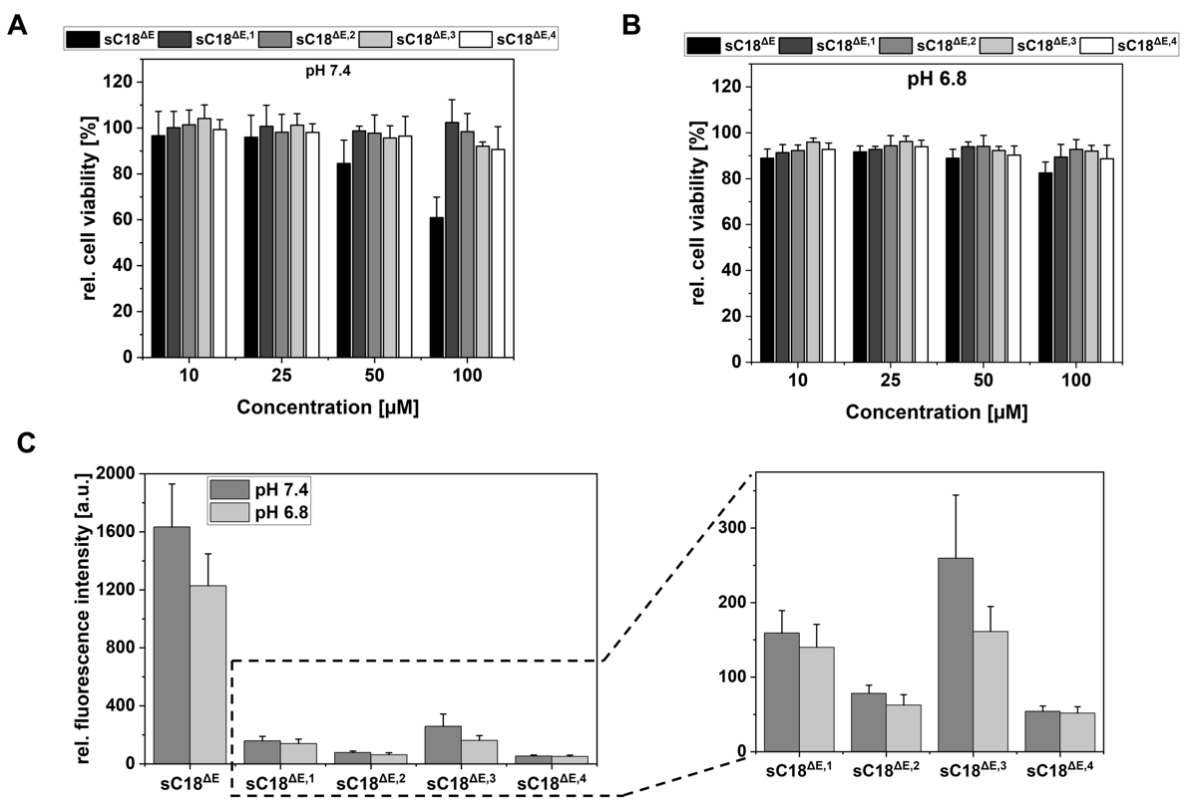
(**A,B**): Cell-viability assays using MCF-7 cells. Cells were cultivated at pH 7.4 or pH 6.8, respectively, for 24 h using different concentrations of peptide solutions. Untreated cells served as negative control; cells treated with 70% ethanol as positive control. Values from the positive control were subtracted from all data, and the untreated cells were set to 100%; assays were conducted in triplicate n = 3. (**C**): Cellular uptake of histidine peptides in MCF-7 cells. Cells were cultivated at different pH-values and incubated with 10 µM peptide solutions for 30 min at 37 °C; assays were conducted in triplicate n = 3.

In the next step, we aimed to elucidate the pH-dependent influence of histidines when peptides were in contact with cells. Therefore, we chose MCF-7 cells (breast cancer cells adenocarcinoma) cultivated at pH 7.4 or 6.8, respectively, and treated them for 24 h with various concentrations of the distinct peptides. While the parent peptide sC18^ΔE^ was slightly more toxic at higher concentrations and when cells were incubated at pH 7.4, all histidine variants displayed no significant differences and no cytotoxicity at all tested concentrations and conditions (Figure 1A,B). Actually, we detected a higher effect on cell viability for sC18^ΔE^ in MCF-7 cells compared to HeLa and HEK293 cells in former studies [12]. Compared to the histidine variants, the herein observed effect might be a result of its higher membrane activity owing to the increased number of Arg and Lys residues. It has been already reported that particularly arginine is a favored amino acid for the interaction with components of the extracellular membrane surface [30]. Although the net charge of the peptide is maintained at acidic conditions, the results shown here let us conclude that arginine and lysine still outcompete histidine concerning this specific membrane interplay. Moreover, MCF-7 might have a membrane composition making them more sensitive toward such membrane-active peptides.

Lastly, we investigated the pH-dependent cellular uptake of the histidine variants in comparison to sC18^ΔE^. Therefore, 10 µM peptide solutions were incubated with MCF-7 cells for 30 min at 37 °C. Flow cytometry analysis revealed a very low uptake of all peptides having histidine included (Figure 1C), compared to sC18^ΔE^. Notably, no significant impact on introduced histidines was recognized since at both pH conditions, the uptake efficiency was nearly comparably low.

In summary, modifying sC18^ΔE^ with histidine did, in our case, not have the expected effect of improved pH-responsiveness and sensitivity; we discarded all these variants from our following studies. On the other side, sC18^ΔE^ seemed to be a promising new candidate for further development as a drug transporter based on its good biocompatibility.

### 2.3. Evaluation of a Shortened sC18 Variant Comprising a Nearly Perfect Amphipathic Helical Structure

Our next investigations focused on the improvement of sC18*. Based on this peptide we created sC18*^R,L^ by exchanging several aa residues with arginine and leucine to receive a nearly perfect amphipathic structure (see Figure S1). Our aim was to enhance the overall uptake efficacy, which was usually relatively low for sC18* itself. Within the next studies, we tested both peptides in comparison to sC18 and sC18^ΔE^. First, we elucidated their cytotoxicity towards HeLa and HEK293 cells. For the peptides sC18, sC18* and sC18^ΔE^ we observed no toxic effect up to 50 µM in HeLa cells, similar to previously obtained results [10,12,25]. Further increasing the concentration of those peptides up to 100 µM resulted in decreased cell viability of up to 75%. On the other side, peptide sC18*^R,L^ had higher activity in HeLa cells compared to the other peptides tested and decreased cell viability at 25 µM to nearly 35%. In HEK293 cells, the toxic effect of sC18*^R,L^ was not that dramatic and started when cells were incubated with 50 µM peptide concentration. The reason for the stronger interaction with HeLa cells might be due to the high content of basic amino acids within the structure of sC18*^R,L^ leading to a strong electrostatically driven approaching of the peptides towards the more negatively charged plasma membrane of HeLa compared to HEK293 cells. This membrane accumulation might then have led to membrane disturbing effects.

**Figure 2:**
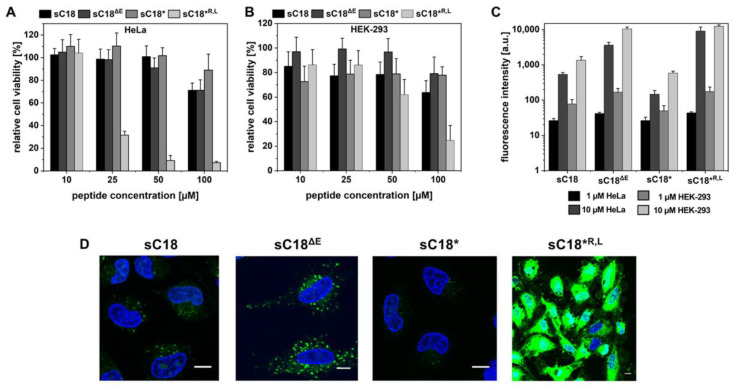
(**A,B**): Cytotoxicity profiles of the peptides in HeLa and HEK-293 cells. Cells were cultured and incubated at pH 7.4 for 24 h with different concentrations of peptide solutions. Untreated cells served as negative control; cells treated with 70% ethanol as positive control. Values from the positive control were subtracted from all data, and the untreated cells were set to 100%; assays were performed with n = 3 in triplicate. (**C**): Flow cytometry analysis of HeLa or HEK-293 cells after incubating 1 and 10 µM peptide solutions for 30 min at 37 °C; assays were conducted in triplicate n = 3. (**D**): Confocal laser scanning microscopy analysis of 10 µM CF-labeled peptides after 30 min of incubation in HeLa cells. Green: CF-labeled peptides; blue: Hoechst 33342 nuclear stain; scale bar is 10 µm.

Next, we compared the uptake potency of sC18 variants in HeLa and HEK293 cells using two different, relatively low concentrations (1 and 10 µM, respectively) to harm the cells as little as possible (Figure 2C). Generally, peptides sC18 and sC18* were taken up by both cell lines to a little extent compared to the other two peptides (sC18^ΔE^ and sC18*^R,L^), while sC18*showed even reduced uptake properties compared to sC18. Moreover, the uptake at 1 µM for sC18^ΔE^ and sC18*^R,L^ was not that drastically increased compared to sC18 and sC18*, but at higher concentrations, a highly significant and enhanced internalization of these peptides took place in both cell lines.

Intracellular accumulation in HeLa cells was verified by confocal fluorescence microscopy and after treating the cells for 30 min with 10 µM peptide solutions, we recognized differences in the uptake patterns. As expected, the general uptake of sC18 and sC18*, respectively, were far lower compared to the other two novel peptides. Furthermore, the uptake mechanisms of sC18^ΔE^ and sC18*^R,L^ appeared to be quite different: sC18^ΔE^ accumulated in a dot-like manner within the cell interior and did not reach the nuclei, while sC18*^R,L^ was highly enriched all over the cytosol including uptake in the nuclei. Thus, we speculated that sC18*^R,L^ interacts with the cell membrane of HeLa cells in a different way compared to sC18^ΔE^ leading to direct penetration instead of an endosomal uptake (Figure 2D).

**Figure 3:**
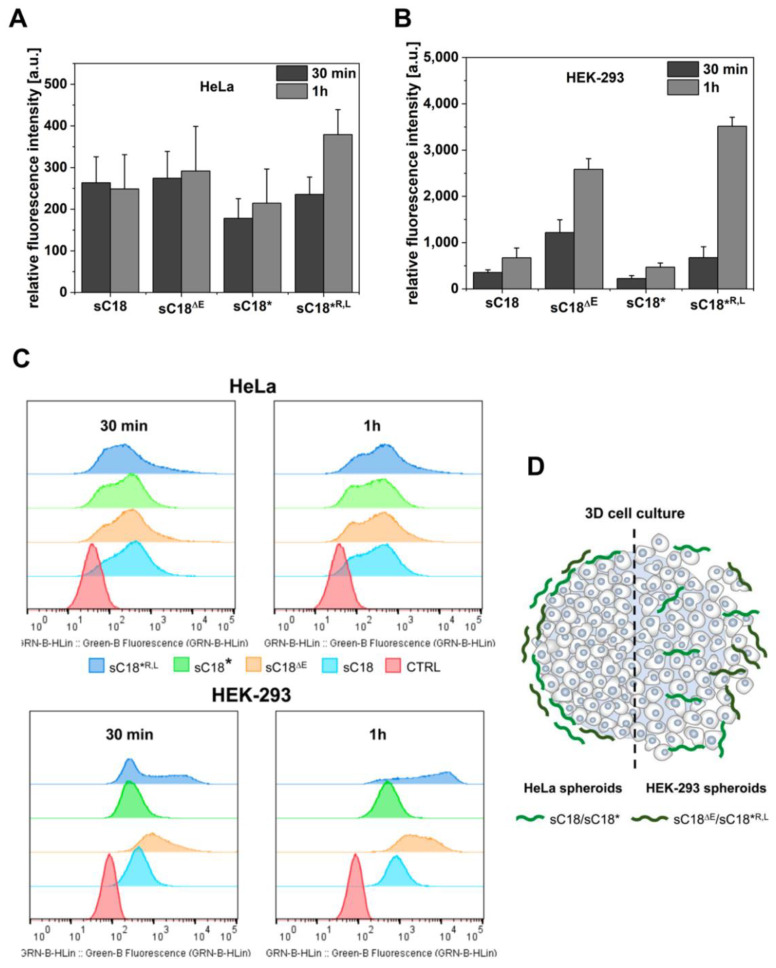
(**A**): Flow cytometry analysis of HeLa spheroids that were incubated with 10 μM CF-labeled sC18 variants for 30 min and 1 h, respectively. (**B**): Flow cytometry analysis of HEK-293 spheroids that were incubated with 10 μM CF-labeled sC18 variants for 30 min and 1 h, respectively. (**C**): Distribution profiles for HeLa and HEK-293 spheroids after 30 min and 1 h incubation time. All experiments were conducted in triplicate with n = 4. (**D**): Proposed distribution profile of sC18-variants in tumor spheroids. Left: Accumulation of sC18-variants in HeLa spheroids. Right: Distribution of sC18-variants in HEK-293 spheroids [35].

Since CPPs were already utilized in many studies as transport systems for anti-cancer drugs [31], we were interested in how the different peptides would perform in a more complex 3D cell system, namely tumor spheroids, which represent a suitable model for the avascular region of tumor tissues [32,33]. Thus, we prepared HeLa as well as HEK-293 spheroids using the hanging drop method [34] and incubated them for 30 min or 1h, respectively, with 10 µM solutions of CF-labeled peptides. As depicted in Figure 3A, the overall accumulation of HeLa spheroids was relatively similar when comparing all sC18 variants with each other. This was surprising, since in the 2D cell model sC18^ΔE^ and sC18*^R,L^ were clearly more active compared to sC18 and sC18*, respectively. Moreover, apart from sC18*^R,L^, increasing the incubation time did not result in enhanced spheroid interaction. Even more surprising were the generally low fluorescence intensity values, compared to the results from the 2D HeLa monolayers, which were much higher for the most potent variant sC18*^R,L^ (see Figure 2D).

Interestingly, in contrast to HeLa spheroids, the results for HEK-293 spheroids were comparable to those obtained for the 2D model, with sC18^ΔE^ and sC18*^R,L^ as the most efficient variants (Figure 3B). Furthermore, the intensities values for all variants increased with a longer incubation time when interacting with HEK-293 spheroids.

To answer the question of why these differences in uptake were observed, we analyzed the distribution profiles of the peptides within the spheroids in more detail (Figure 3C). While the population of the untreated controls showed a uniform Gaussian distribution profile for both tested cell lines and incubation times, all samples treated with sC18 variants appeared in a much broader curve for HeLa spheroids after 30 min as well as after 1 h incubation. As recently reported, the broader distribution would rather refer to peptides that penetrated relatively little and were more embedded within the peripheral region of the spheroid [35]. The reason behind this might be the overall more negatively charged membrane composition of HeLa cells being responsible for higher electrostatic attractions following increased accumulation within the peripheral region of the spheroid. In contrast to this, the histograms of the HEK-293 spheroids illustrated a uniform distribution for sC18 and sC18* for both incubation times (Figure 3C), while for sC18^ΔE^ and sC18*^R,L^ a wider distribution was detectable. This led to the conclusion that those variants were not homogeneously distributed within the spheroid. One explanation for this phenomenon might be that the penetration depth of CPPs within such cell models is supposed to be dependent on their membrane activity [35]. For instance, for the CPPs penetratin and nona-arginine, it was reported that the more amphiphilic penetratin was most likely to accumulate at the outer layer of the spheroid, whereas nona-arginine was able to diffuse in the interstitial space of the spheroid [35]. Taking into account that sC18^ΔE^ and sC18*^R,L^ are presumably more membrane-active, due to their pronounced more balanced amphipathic character, it is possible that especially those variants accumulated in the peripheral region of the tumor spheroid, not reaching the core efficiently. However, both variants are also rich in arginine; therefore, it is possible that a combination of both enrichment on the outer surface of the spheroid, as well as diffusion to the spheroid core, occurred during the interaction process, leading to the broad distribution profile (Figure 3D).

**Figure 4:**
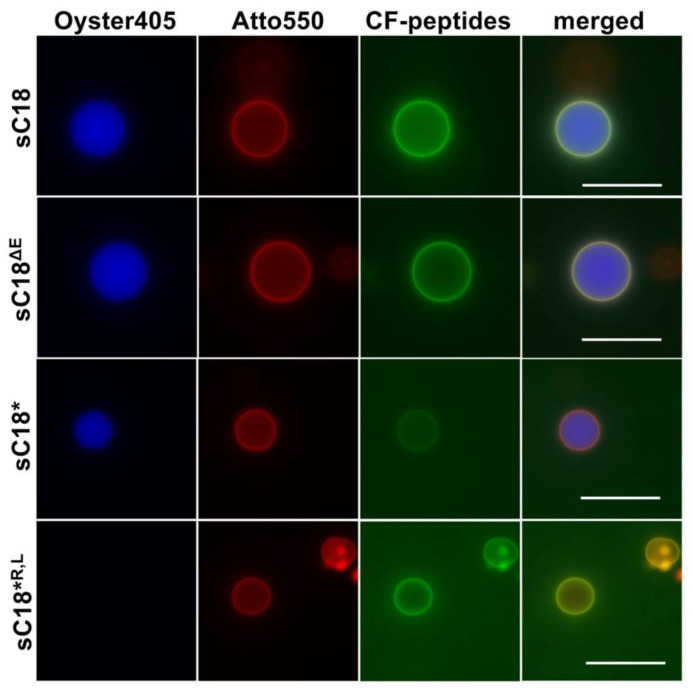
Peptide interaction with giant lamellar vesicles (GUVs) composed of DOPC/DOPE/DOPG (40:30:30); 1 µM solutions of sC18 variants were incubated with GUVs for 30 min and inspected using a fluorescence microscope (Keyence). Red: Atto550; green: CF-labeled peptides, blue: Oyster 405. Scale bar: 50 µm.

In order to further determine the differences in membrane activity, we conducted lipid-peptide interaction studies using anionic giant unilamellar vesicles (GUVs) mimicking cancerous membrane compositions, as well as zwitterionic GUVs representing healthy cells [16]. Both types of vesicles were treated with 1 µM of sC18 variants for 30 min. We chose to use the lower concentration due to the observed strong internalization efficacy of sC18*^R,L^. All peptides were able to accumulate onto the surface of anionic vesicles, but it seemed that the fluorescence signal of sC18* was weaker compared to the other peptides (Figure 4). Particularly, sC18*^R,L^ showed the strongest accumulation leading to membrane disruption events and complete dye outflow from the vesicles. On the other side, at this low concentration, we did not observe any interaction with the zwitterionic vesicles (Figure S11). However, this strong interaction with the negatively charged GUVs matched our previous results [16] and let us conclude that sC18*^R,L^ is a highly membrane-active peptide. This effect is assumedly a consequence of the well-balanced amphipathic helical character leading to strong interactions with the cell membrane.

From our results thus far, we reasoned that the novel sC18-variants, sC18^ΔE^ and sC18*^R,L^, efficiently internalize into HeLa and HEK-293 cells, but presumably via different uptake mechanisms. In principle, the elimination of glutamate leading to peptide sC18^ΔE^, as well as optimizing the hydrophobic part resulting in peptide sC18*^R,L^ turned out as powerful strategies to improve the performance of the CPP sC18. Both peptides demonstrated a higher cellular uptake compared to their parent variants sC18 and sC18*. However, we claim that sC18^ΔE^ might be the more suitable candidate for future applications in drug delivery, since it demonstrated an overall good cellular uptake while exhibiting far lower cytotoxic activity compared to sC18*^R,L^.

### 2.4. Synthesis of Peptide-Drug Conjugates (PDCs) and Their Biological Evaluation

**Scheme 1:**
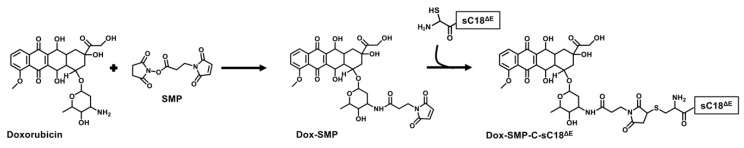
Synthesis scheme of Dox-SMP-peptide conjugates. Exemplarily, the synthesis of PDC-2 is shown (see also Table 2 for peptide sequence).

Based on the fact that sC18*^R,L^ exhibited strong membrane activity and cytotoxicity (Figure 2, Figure 3 and Figure 4), we decided to conduct the next experiments using sC18, sC18^ΔE^ and sC18* only. To compare these peptides in their performance to act as drug delivery vehicles, we generated peptide-drug conjugates and selected, as proof-of-principle, the anti-cancer drug doxorubicin. Indeed, the clinical use of doxorubicin is still hampered by its dose-limiting toxicity related to several appearing side effects [36,37]. Therefore, coupling to a CPP might be beneficial in terms of biocompatibility. Scheme 1 displays the general synthesis strategy to obtain the distinct PDCs.

Within the first step, we modified doxorubicin with the bifunctional linker *N*-succinimidyl-3-maleimidopropionate (SMP), while monitoring the reaction progress by thin layer chromatography (TLC). Meanwhile, the respective sC18 variant was extended *N*-terminally by an additional cysteine to enable a covalent reaction with the maleimide moiety of the SMP linker. In the last step, both components were coupled via a Thiol–Michael addition to yield PDC-1, PDC-2 and PDC-3, having incorporated the peptides sC18, sC18^ΔE^ and sC18*, respectively (Table 2 and Figures S12–S14).

CD spectra of the new conjugates showed that the secondary structure of the peptides was not affected (Figure S15). As already mentioned, this is a prerequisite for membrane interaction, which we assume takes place via electrostatic interaction with the positively charged residues of the amphipathic helix of the CPP.

**Figure 5:**
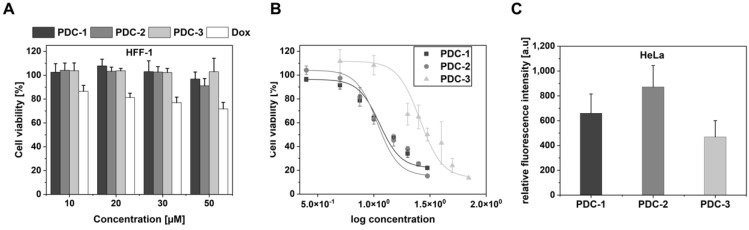
(**A**): Cytotoxicity assay using HFF-1 cells. Different PDC concentrations were incubated with the cells for 24 h at 37 °C and a resazurin assay was conducted. (**B**): Dose-response curves for peptide drug conjugates were obtained using the resazurin assay. For this, HeLa cells were treated with various conjugate concentrations (2.5–70 µM) for 24 h at 37 °C. (**C**): Quantification of PDC internalization in HeLa cells; 10 µM PDC solutions were incubated with cells for 30 min at 37 °C and cells were inspected using a flow cytometer; assays were conducted in triplicate n = 3.

First, we probed the novel PDCs in non-cancerous human foreskin fibroblasts (HFF-1 cells). After 24 h treatment with different concentrations of PDCs, HFF-1 cells were still viable, while after adding doxorubicin viability was decreased up to 72% (Figure 5A).

**Figure 6:**
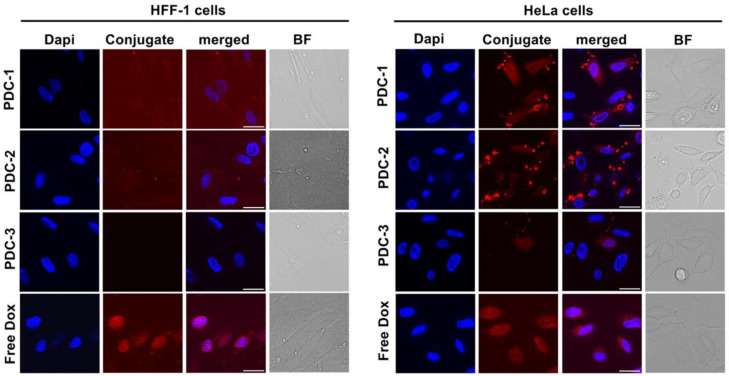
Fluorescence microscope analysis of 5 µM conjugates after 30 min of incubation at 37 °C in HeLa and HFF-1 cells. Red: peptides coupled to red fluorophore Doxorubicin; blue: Hoechst 33342 nuclear stain; BF: brightfield to identify living cells; scale bar is 20 µm.

In comparison, when we elucidated PDC activity in HeLa cells and exposed them for 24 h to various concentrations of the conjugates (2.5–70 µM) (Figure 5B), all of the PDCs, as well as free Dox, exhibited EC_50_ values in the lower micromolar range (PDC-1: 15.34 µM, PDC-2: 14.47 µM, PDC-3: 27.01 µM, Dox: 6.78 µM data not shown). The higher activity compared to HFF cells might be attributed to the fact that the PDCs were internalized to far less of an extent into the non-cancerous cell line (Figure 6). This observation might be advantageous and could reflect some selectivity of the more basic and positively charged peptides towards cancerous cells. We also noted that the obtained EC_50_ values somehow agreed with the results of the former assays. For example, sC18^ΔE^ was taken up to a significantly higher extent compared to sC18* and should, therefore, exhibit higher activity, e.g., drug delivery. However, surprisingly, the EC_50_ values of the PDCs containing sC18 and sC18^ΔE^ were quite similar, although sC18^ΔE^ significantly outcompeted sC18 in internalization activity (Figure 2).

Therefore, we performed internalization studies taking advantage of the red fluorescence of doxorubicin. We incubated HeLa cells for 30 min with 5 µM solutions of the respective PDC and quantified the uptake using flow cytometry (Figure 5C). Although the uptake of PDC-2 seemed to be enhanced compared to the other two, particularly compared to PDC-1 having sC18 included, the difference was not as high as we observed before (Figure 2C). Moreover, when we inspected the cells by fluorescence microscopy, we realized that PDC-2 is somehow clustering inside the cytosol and that probably, PDC-1 was more efficiently released after endosomal uptake, so more fluorescence was also measured within the nuclei. Indeed, it might be that sC18^ΔE^ is more efficiently internalized; however, its uptake may take place mainly via endocytosis leading to the entrapment of the cargo–peptide conjugate. As a result, strategies to enhance the release of the peptide–cargo construct will be evaluated by us in future studies.

## 3. Materials and Methods

### 3.1. Materials

N_α_-Fmoc protected amino acids were purchased from IRIS Biotech (Marktredwitz, Germany). Other chemicals and consumables including 1-bis(dimethylamin)methylen]-1H-1,2,3-triazol[4,5-b]pyridinium-3-oxide hexafluorophosphate (HATU), N,N-diisopropylethylamine (DIPEA), acetonitrile (ACN), and trifluoroacetic acid (TFA), doxorubicin hydrochloride (Dox), N-succinimidyl-3-maleimidopropionate (SMP), N,N′-diisopropylcarbodiimide (DIC), triethanolamine (TEA), 5(6)-carboxyfluorescein (CF) were derived from Fluka (Taufkirchen, Germany), Merck (Darmstadt, Germany), Sarstedt (Nümbrecht, Germany), Sigma-Aldrich (Taufkirchen, Germany) and VWR (Darmstadt, Germany).

The lipids for GUV formation were purchased from Avanti Polar Lipids, Inc. (Alabaster, AL, USA). Atto550 labeled DOPE was purchased from Atto Tec (Siegen, Germany).

### 3.2. Solid Phase Peptide Synthesis

All peptides were synthesized on Rink amide resin by automated SPPS on a multiple Syro II peptide synthesizer (MultiSyntech, Witten, Germany) following Fmoc/*t*Bu-strategy as recently described. Briefly, amino acids were coupled using a double-coupling procedure and in situ activation with Oxyma/DIC. Purification of peptides was achieved by preparative reverse phase HPLC on a C18 column and analyzed by analytical HPLC ESI-MS (LTQ XL, Thermo Scientific, Waltham, MA, USA). Purified peptides were evaporated and lyophilized with purities > 97%. All peptides were also synthesized as 5,6-carboxyfluorescein (CF) variants by coupling CF to the *N*-terminal of the peptides while still being attached to the solid support.

### 3.3. PDC Synthesis

Doxorubicin (dox) was conjugated to the different sC18-derivates via an SMP linker. First, doxorubicin was coupled to *N*-succinimidyl-3-maleimidopropionate (SMP) using triethanolamine (TEA). Thus, dox, SMP and TEA were dissolved in DMF with a molar ratio of 1.1:1:2 and stirred in the dark for at least 3 h. The coupling process was monitored by thin-layer chromatography (TLC) using chloroform, methanol and ammonia as the liquid phase (70:30:3, (*v*/*v*/*v*)). Afterward, the reaction mixture was precipitated in ice-cold diethylether and incubated overnight at −20 °C. The solution was then centrifuged several times at 5000× *g* for 5 min at 4 °C and resuspended again in diethylether and dried. Subsequently, Cys-sC18 and dox-SMP were dissolved in DMF and mixed in a molar ratio of 1.5:1 (dox-SMP:peptide). The reaction was stirred in the dark at room temperature for 48 h and monitored by TLC as described before. Finally, the reaction solution was concentrated and precipitated in ice-cold diethylether overnight. Then, the conjugates were purified using preparative RP-HPLC (Nucleodur C18ec; 100-5; Macherey-Nagel, Düren, Germany) and analyzed by HPLC ESI-MS as described above.

### 3.4. Circular Dichroism (CD) Spectroscopy

CD spectra were recorded using a JASCO J-715 spectropolarimeter (JASCO, Pfungstadt, Germany) in an N_2_ atmosphere. The CD spectra were measured from 180 nm to 270 nm in 0.5 nm intervals at 20 °C using a 1 mm quartz cuvette and the instrument parameters were set as follows: sensitivity, 100 mdeg; scan mode, continuous; scan speed, 50 nm/min; response time, 2 s and bandwidth, 2.0 nm; 10 µM peptide solutions in 10 mM potassium phosphate buffer (pH 7.0) were inspected containing either 0 or 50% (*v*/*v*) trifluoroethanol (TFE).

### 3.5. Peptide Interaction with Giant Unilammellar Vesicles (GUVs)

A thin agarose layer is necessary to build GUVs. Therefore, 1% super low melting agarose was melted in deionized water by heating up in a microwave. Afterward, 200 µL were coated on a clean slide and dried at 50 °C for 30 min on a heating plate; 10 µL of respective lipid solution was added onto the agarose layer and subsequently distributed and dried for 1 h using an exicator under vacuum to get rid of the chloroform. For staining the lipid layer, 0.2 mol % Atto550 was added. Afterward, a sealing ring was added around the pink lipid film. Lipids were hydrated with dextran buffer (10 mM HEPES buffer (pH 7.4), 50 mM KCl, 50 mM NaCl and 1 mg/mL Dextran) containing 3 µL Oyster405 (Luminaris GmbH, Münster, Germany) encapsulating the blue dye. Slides were incubated for 2 h at RT. Afterward, lipids were transferred into fresh PCR tubes and centrifuged for 10 min at maximum speed. The supernatant was removed, and the pellet was resuspended in 300 µL dextran buffer. For fluorescence microscopy analysis, lipids were transferred into an eight-well Ibidi^®^ plate; 40 µL of respective GUV solution was mixed with 1 µM peptide solutions and filled up to 100 µL with dextran buffer. Incubation with peptides was performed for 30 min at room temperature. Afterward, GUVs were analyzed using a BZ-X800E microscope (Keyence, Osaka, Japan) followed by image processing with ImageJ.

### 3.6. Cell Culture

Cell lines HEK-293 (human embryonic kidney, ACC305), MCF-7 (human adenocarcinoma, Dr. Jozsef Tovari (Institute of Oncology, Budapest)) cells, HFF-1 (human foreskin fibroblasts, ATCC), as well as HeLa (human cervix carcinoma, ACC57) cells were used. HEK-293 cells were cultured in MEM (M2279) medium supplemented with 4 mM L-glutamine and 15% FBS (fetal bovine serum). HeLa and MCF-7 cells were cultured in RPMI-1640 (R0883) medium supplemented with 4 mM L-glutamine and 10% FBS. HFF-1 cells were cultured in flasks containing DMEM (D5030) medium supplemented with 4 mM L-glutamine and 10% FBS. All cell lines except for the HFF-1 were cultured in 10 cm sterile Petri dishes at 37 °C and 5 CO_2_ in a humidified atmosphere.

For pH-dependent studies, the cells were cultivated and incubated at pH 6.8 or 7.4, respectively.

### 3.7. Seeding and Treatment of Tumor Spheroids

For the preparation and seeding of spheroids, cells were washed and trypsinized as described above. Cells were transferred into a fresh 15 mL centrifuge tube and centrifuged for 5 min at 300× *g* and rt. The supernatant was removed carefully and the pellet was resuspended in an appropriate medium supplemented with FBS. Cells were counted and a cell mixture was prepared, which would be sufficient for 100 drops (3 mL). Within this mixture a total amount of 1.5 × 10^6^ cells (HEK-293 and HeLa cells) and 20% (*v*/*v*) methylcellulose (1.2 mg/mL) were required. The missing volume was filled up with an appropriate medium containing FBS; 30 μL drops were pipetted into the inverted lid of a petri dish (15,000 cells per drop), the bottom was covered with PBS and spheroids were grown in hanging drops for two or three days at 37 °C and 5% CO_2_, respectively.

### 3.8. Flow Cytometry Studies

Monolayer cellular uptake was quantified using flow cytometry. Cells were seeded onto a 24-well plate (HeLa: 90,000, HEK293: 100,000 and MCF-7 130,000 cells per well) and grown to 80–90% confluency. Then, cells were incubated with the labeled peptides/conjugates in a serum-free medium at 37 °C for various incubation times, and subsequently washed twice with PBS and detached with indicator-free trypsin resuspended in an indicator-free serum-containing medium. The cell suspension was transferred into a 96-well FACS plate and fluorescence was then measured by a Guava easyCyte flow cytometer (Merck, Darmstadt, Germany).The blue laser was used for all measurements, FSC (1137–1538), SSC (3020–4086), Grn-B (647–875) and Red-B (694–939). Cellular autofluorescence of untreated control cells was subtracted.

For flow cytometry studies with tumor spheroids, the spheroids were prepared and grown as described above (4.7). CF-labeled peptides were diluted in an appropriate serum-free medium and 3 μL were pipetted into the surrounding drop of the spheroid. Typically, ten spheroids per condition were chosen and the peptides were incubated for 30 min or 1 h at 37 °C and 5% CO_2_, respectively. After incubation time, spheroids were harvested and transferred into a fresh reaction vessel. The spheroids were allowed to settle and the remaining medium was carefully removed. Subsequently, they were washed twice with 200 μL PBS. After the addition of 150 μL trypsin-EDTA (indicator free), spheroids were gently shaken for 5 min at 37 °C in a Thermomixer. After the spheroids started to fall apart, 850 μL of medium with FBS (indicator free) was added and the spheroids were resuspended to generate a homogenous cell suspension; 200 μL of each sample were transferred into a fresh round-bottom 96-well plate and analyzed on a Guava^®^ easyCyte flow cytometer (Merck, Darmstadt, Germany). The blue laser was used for all measurements, FSC (1137–1538), SSC (3020–4086), Grn-B (647–875) and Red-B (694–939). Cellular autofluorescence of untreated control cells was subtracted.

### 3.9. Microscopy Analysis

Cells were seeded into 8-well plates (HeLa cells 35,000 and HFF-1 25,000 cells per well, respectively) and grown to 80–90% confluency. Cells were treated with 10 μM CF- or dox-labeled peptides in a serum-free medium for 30 min at 37 °C. Ten minutes prior to the end of the incubation time, nuclei were stained with Hoechst33342. The peptide solution was removed, and cells were washed five times with PBS and covered with fresh medium including FBS. Microscopic analysis was performed using an inverse confocal TCS SP8 microscope (Leica Microsystems, Wetzlar, Germany), equipped with a 63× oil-immersion objective. Images were recorded with LAS X software (LAS_X_Core_3.7.6_25997, Leica Microsystems, Wetzlar, Germany). For the GUV analysis and the uptake of the peptide-drug conjugates the Keyence fluorescence tabletop microscope BZ-X800E (Keyence, Osaka, Japan), with a 60× oil immersion lens was used. Images were recorded with Keyence software (BZ-800X_Analyzer 1.1.1.8, Keyence, Osaka: Japan) and evaluated with Fiji.

### 3.10. Cytotoxicity Assay

For cell viability assay, cells were (15,000 HeLa, 17,000 MCF-7 and 12,000 HFF-1 cells per well) seeded onto a 96-well plate and grown to 80–90% confluency. Cells were incubated with several peptide concentrations in a serum-free growth medium for 24 h under standard growth conditions. For the positive control, cells were treated with 70% EtOH for 7 min. After washing with DPBS, resazurin solution (10% in serum-free media, *v*/*v*) was incubated with the cells for 1 h under standard growth conditions. The cell viability was determined relative to untreated cells by measurement of the resorufin product at 595 nm (λex = 550 nm) on a Tecan infinite M200 plate reader (Tecan Group AG, Männedorf, Switzerland).

## 4. Conclusions

In conclusion, we presented the design, synthesis and characterization of different variants of the cell-penetrating peptide sC18. sC18 has been used in various studies as a carrier peptide; however, recent structure-activity relationship studies have revealed several starting points of optimization. For instance, eliminating the glutamate in the *C*-terminal part yielded a CPP with significantly higher internalization abilities [12]. Thus, we investigated the impact of substituting several amino acids with histidine within this peptide, since it was recently discussed to improve pH-responsiveness on several levels. Unexpectedly, we did not gain any supportive effect from the presence of histidine. Since we generated several different histidine mutants, we are convinced that this effect is not related to the importance of specific amino acid positions within the sequence. We hypothesize that our finding may be more of a general characteristic of the sC18 sequence. In fact, we already know that the formation of the amphipathic helix is quite important, as well as a balanced ratio of basic and hydrophobic amino acids. As a consequence, for two of the histidine variants, the obtained results might be the effect of a decreased hydrophobic moment that we know plays a critical role in successful and efficient membrane interaction (see Table 1). At the same time, the presence of arginine residues with its guanidine group might be favored for membrane activity owing to its improved ability to interact with chemical groups at the extracellular side of the plasma membrane compared to histidine with the imidazole ring.

On the other side, increasing this amphipathicity generated peptides with far too high membrane activity, as was shown for sC18*^R,L^. Although very promising in uptake efficiency, we discarded this peptide from further studies owing to its high cytotoxicity. However, the fact that such highly membrane-active peptides often comprise antimicrobial activity, will place our focus in this direction when we establish this peptide in the future. Finally, creating PDCs containing the different variants of sC18 supported the previously found good drug delivery performance of those peptides. Additionally, we identified sC18^ΔE^ as highly efficient and are convinced that we can improve its potency as a transport vehicle by, e.g., the introduction of specific cleavage sites between the drug and carrier, as we have already successfully demonstrated in former experiments [15].

## Figures and Tables

**Table 1:** Names, sequences, molecular weights and hydrophobic moments (µH^a^) of synthesized peptides. Peptides were generated as *C*-terminal amides.

Name	Sequence	MW_calc._ [Da]	MW_exp._ [Da]	*µ*H
sC18^ΔE^	GLRKRLRKFRNKIKK	1940.44	1941.04	0.656
sC18^ΔE1^	GLRHRLRHFRNHIHH	1985.27	1986.02	0.465
sC18^ΔE2^	GLHKHLHKFHNKIKK	1864.25	1864.92	0.667
sC18^ΔE3^	HLRHHLRHFRHHIKH	2060.38	2061.17	0.524
sC18^ΔE4^	GLHKRLHKFHNKIHK	1892.26	1893.00	0.655
sC18*	GLRKRLRKFRNK	1570.94	1571.33	0.572
sC18*^R,L^	RLRKLLRKFLRK	1626.10	1626.46	0.790
sC18	GLRKRLRKFRNKIKEK	2069.55	2069.87	0.555

^a^ https://heliquest.ipmc.cnrs.fr (accessed on 1 August 2022).

**Table 2:** Names, sequences and molecular weights of synthesized peptide-drug conjugates, PDC-1, PDC-2 and PDC-3, having coupled doxorubicin at the *N*-terminal via an SMP linker.

Name	Sequence	MW_calc._ [Da]	MW_exp._ [Da]
PDC-1	*Dox*-GLRKRLRKFRNKIKEK	2868.43	2868.03
PDC-2	*Dox*-GLRKRLRKFRNKIKK	2738.31	2738.90
PDC-3	*Dox*-GLRKRLRKFRNK	2368.81	2368.95

## Data Availability

The data presented in this study will be available upon request.

## Supplementary Materials

The following supporting information can be downloaded at: https://www.mdpi.com/article/10.3390/molecules27196656/s1, Figures S1–S15 including helical wheel projections, analysis of all synthesized peptides and PDCs, CD spectroscopy measurements and experiments using zwitterionic GUVs.

## Sample Availability

Samples of the compounds are available upon request.
