# Supplementary material for: Comparing Variants of the Cell-Penetrating Peptide sC18 to Design Peptide-Drug Conjugates
Source: Molecules. 2022 Oct 7;27(19):6656. doi: 10.3390/molecules27196656 (PMC9570898; doi:10.3390/molecules27196656)
Supplement: Supplementary file 1 [file molecules-27-06656-s001.zip › molecules-1910388-supplementary.pdf]

Supplementary Information

Comparing variants of the cell-penetrating peptide sC18 to design peptide-drug conjugates

Joshua Grabeck, Tamara Lützenburg, Pia Frommelt, Ines Neundorf

University of Cologne, Faculty of Mathematics and Natural Sciences, Department of Chemistry, Biochemistry, Zuelpicher Str. 47a, 50674 Cologne, Germany

Corresponding author: Prof. Dr. Ines Neundorf; ines.neundorf@uni-koeln.de

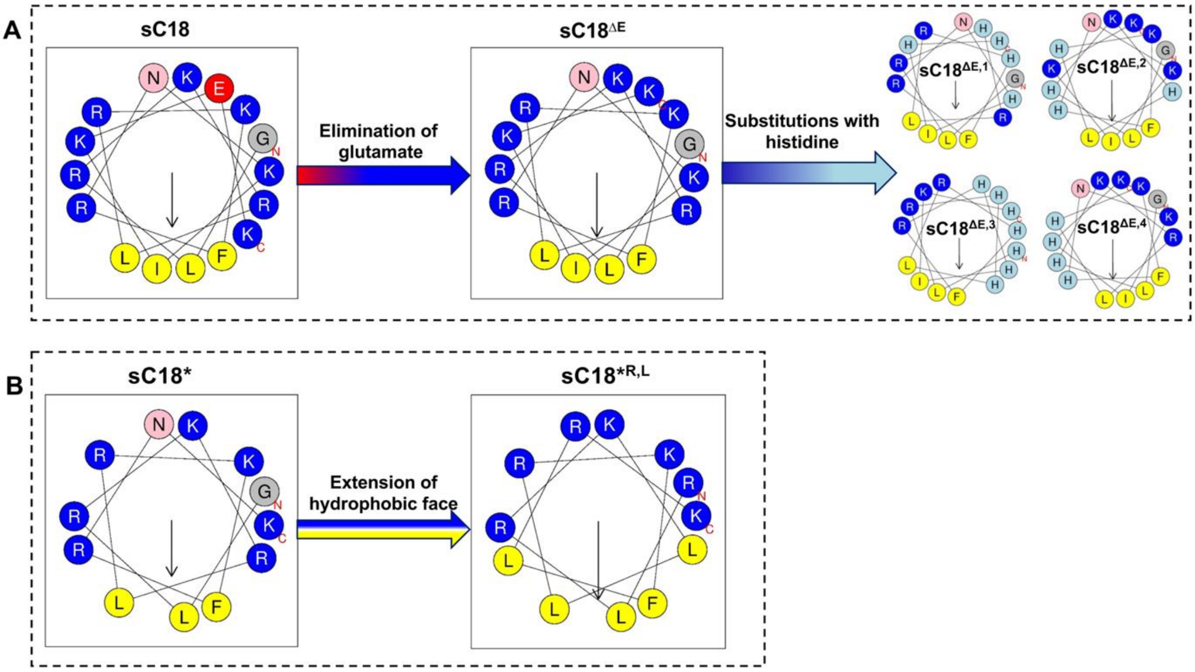

Figure S1 Peptides investigated within this work were based on two different sC18 variants **(A)** sC18$^{\Delta E}$ lacks glutamate and is, thus, more hydrophilic. To increase pH-responsiveness, variants sC18$^{\Delta E1\text{-}4}$ were generated having different substitutions with histidine included. **(B)** sC18* lacks four amino acids at the C-terminal part. Within this study, two more arginines were introduced by substituting glycine and asparagine. This leads to a nearly perfect amphipathic helix.

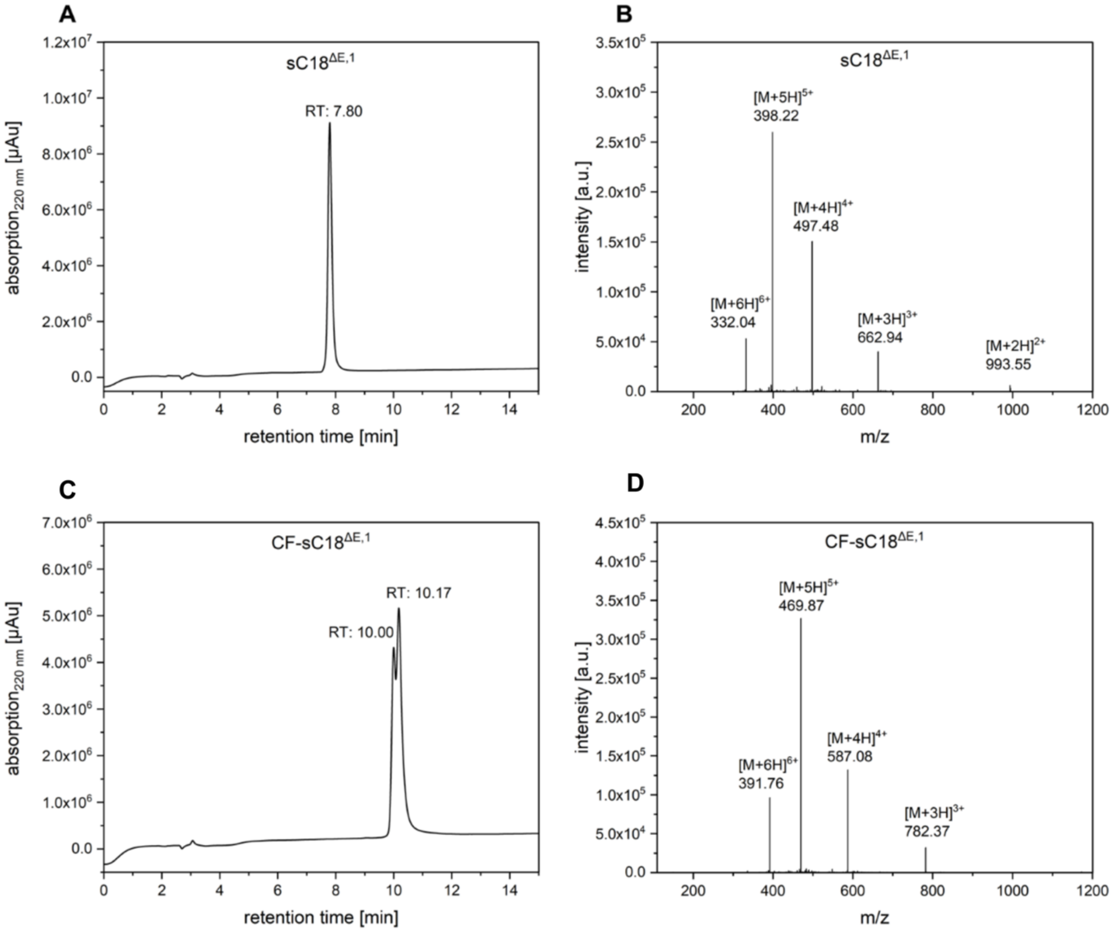

Figure S2 LC-MS analysis showing UV chromatographs was recorded using a linear gradient from 10–60% ACN in water (incl. 0.1% TFA) over 15 min (A, B). Mass spectra of sC18$^{\Delta E1}$ and CF-sC18$^{\Delta E1}$ (C, D), respectively.

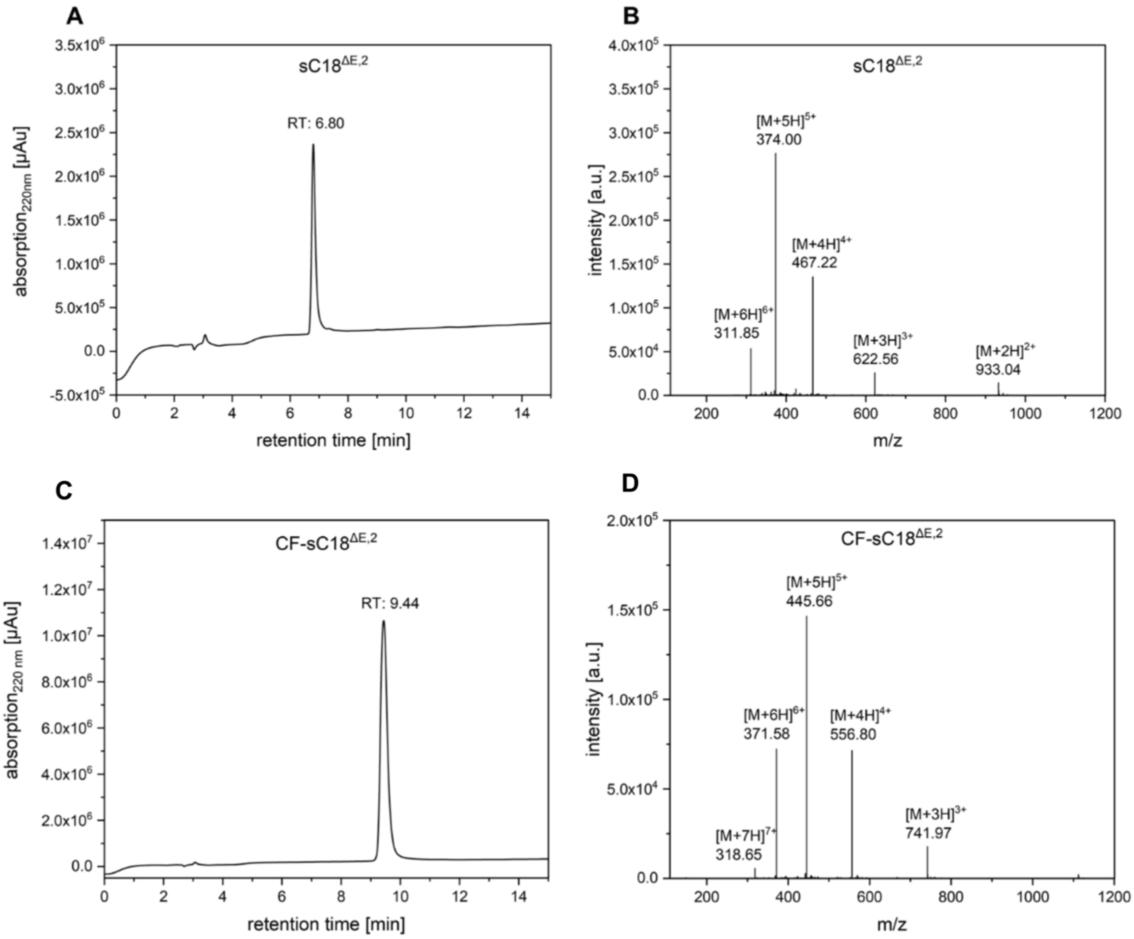

Figure S3 LC-MS analysis showing UV chromatographs was recorded using a linear gradient from 10–60% ACN in water (incl. 0.1% TFA) over 15 min (A, B). Mass spectra of sC18$^{\Delta E2}$ and CF-sC18$^{\Delta E2}$ (C, D), respectively.

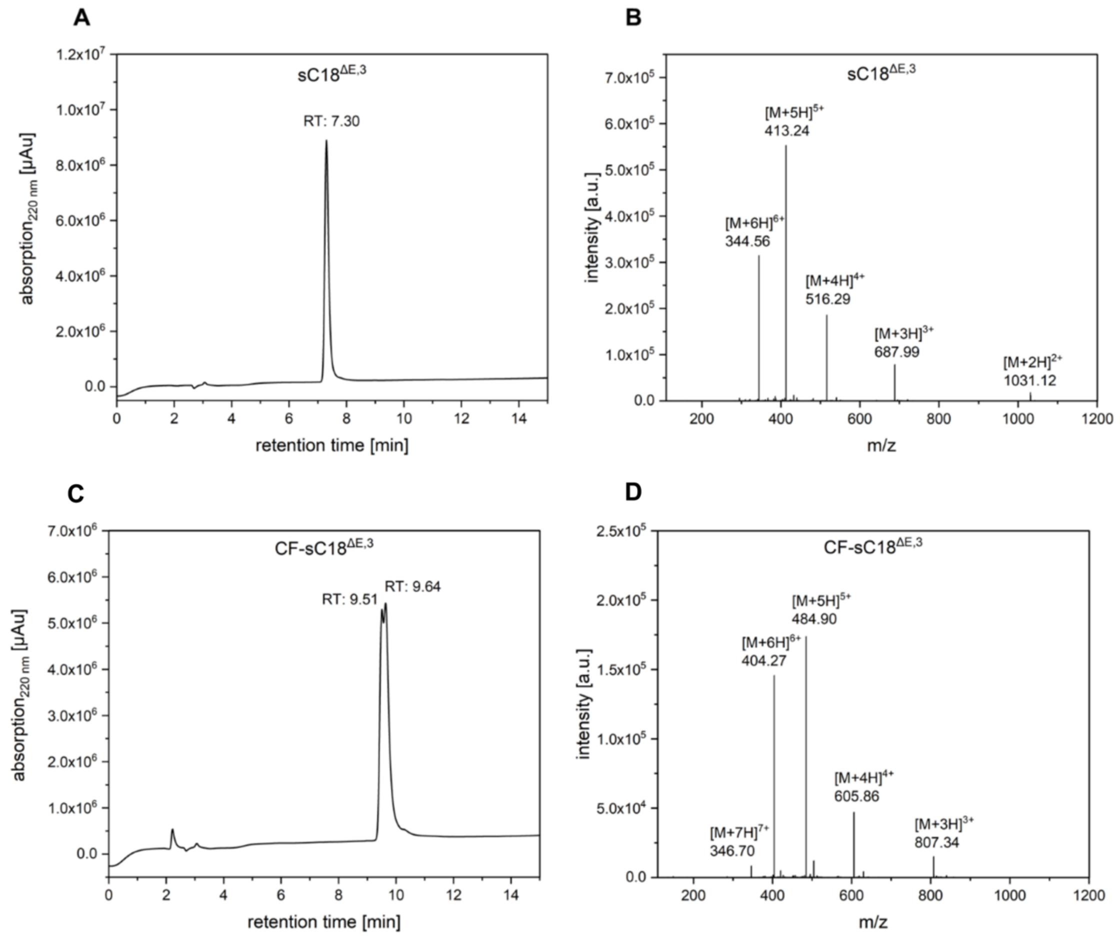

Figure S4 LC-MS analysis showing UV chromatographs was recorded using a linear gradient from 10–60% ACN in water (incl. 0.1% TFA) over 15 min (A, B). Mass spectra of sC18$^{\Delta E3}$ and CF-sC18$^{\Delta E3}$ (C, D), respectively.

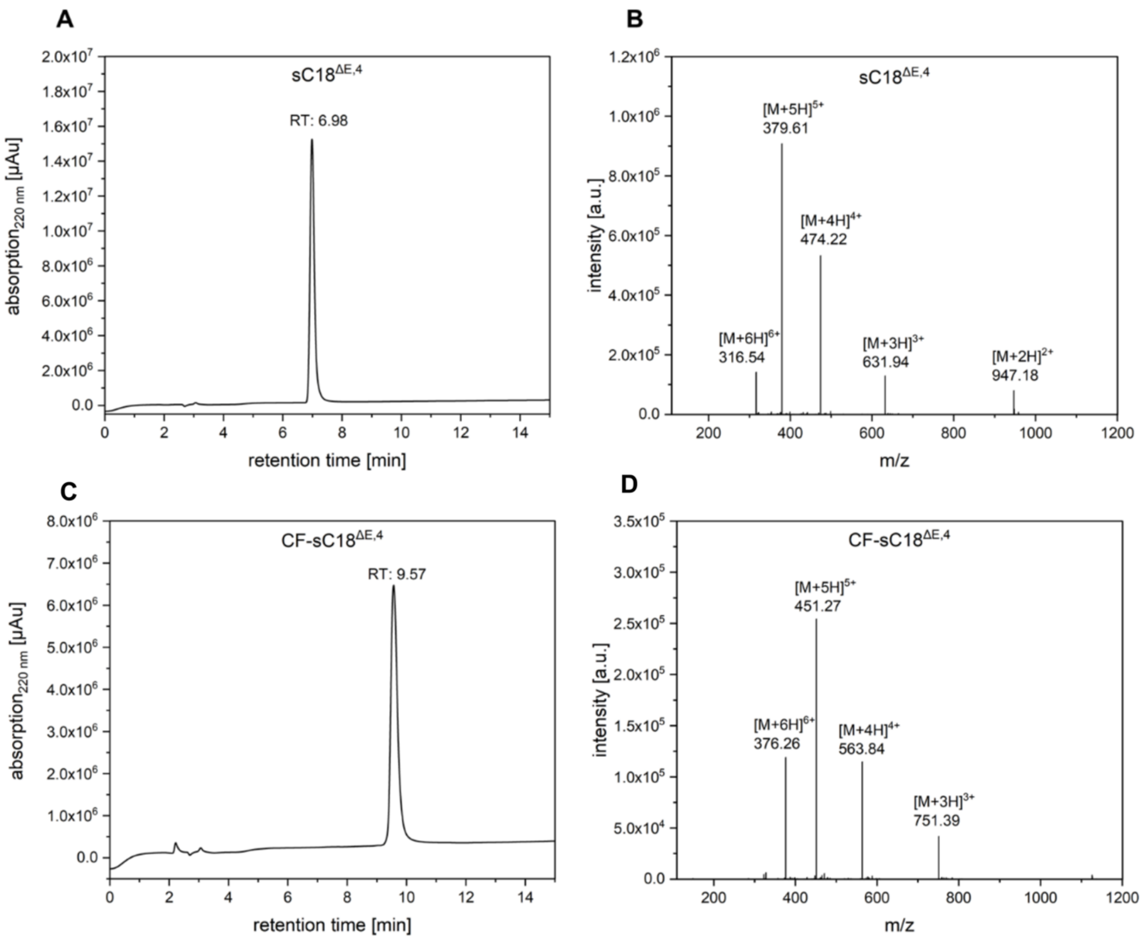

Figure S5 LC-MS analysis showing UV chromatographs was recorded using a linear gradient from 10–60% ACN in water (incl. 0.1% TFA) over 15 min (A, B). Mass spectra of sC18$^{\Delta E4}$ and CF-sC18$^{\Delta E4}$ (C, D), respectively.

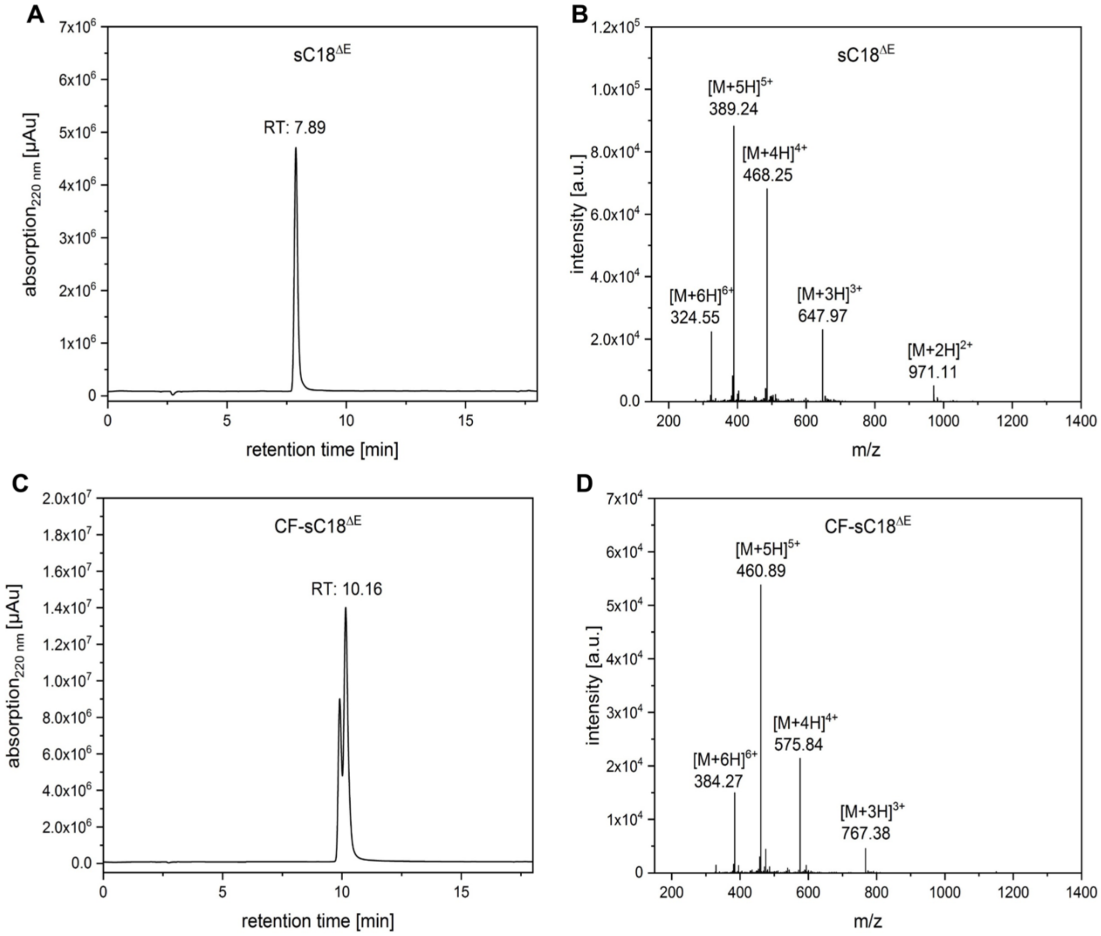

Figure S6 LC-MS analysis showing UV chromatographs was recorded using a linear gradient from 10–60% ACN in water (incl. 0.1% TFA) over 15 min (A, B). Mass spectra of sC18$^{\Delta E}$ and CF-sC18$^{\Delta E}$ (C, D), respectively.

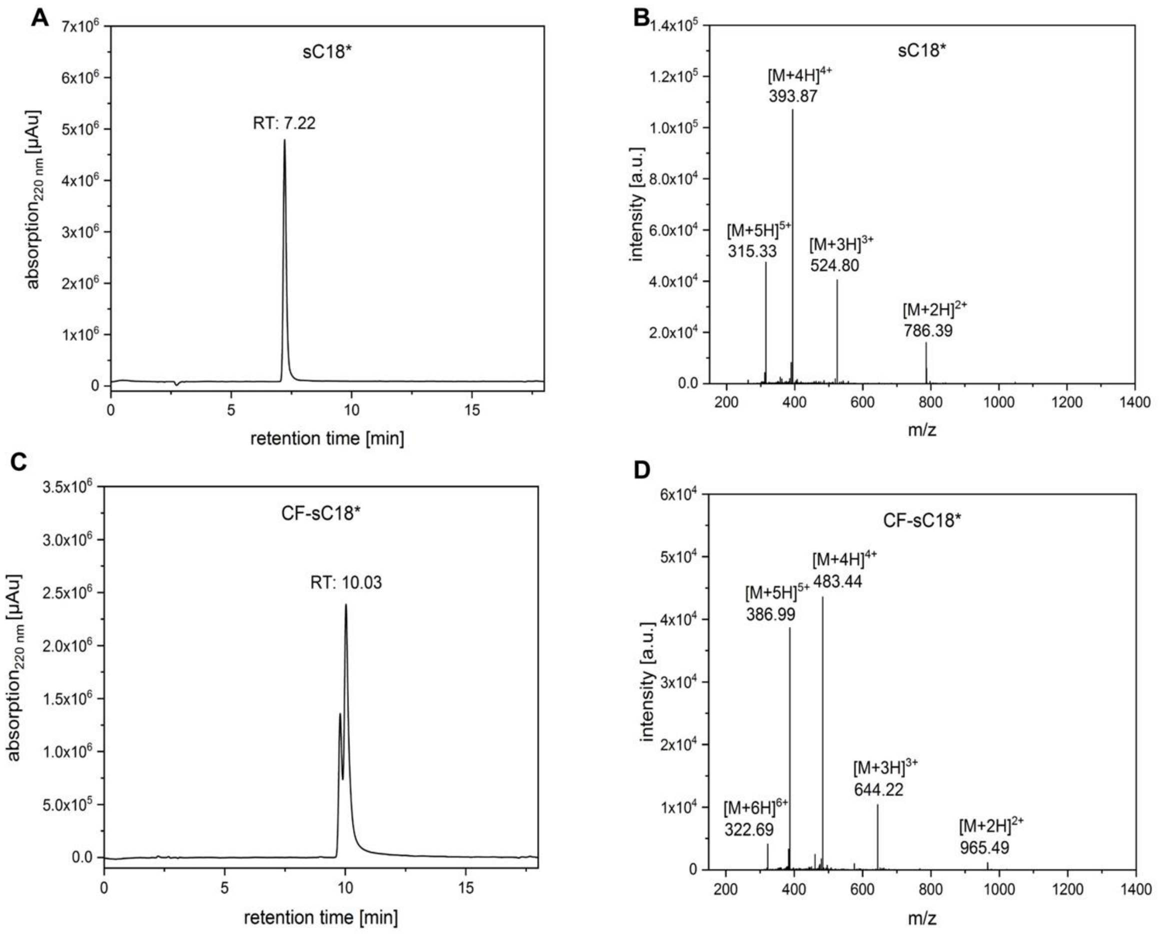

Figure S7 LC-MS analysis showing UV chromatographs was recorded using a linear gradient from 10–60% ACN in water (incl. 0.1% TFA) over 15 min (A, B). Mass spectra of sC18* and CF-sC18* (C, D), respectively.

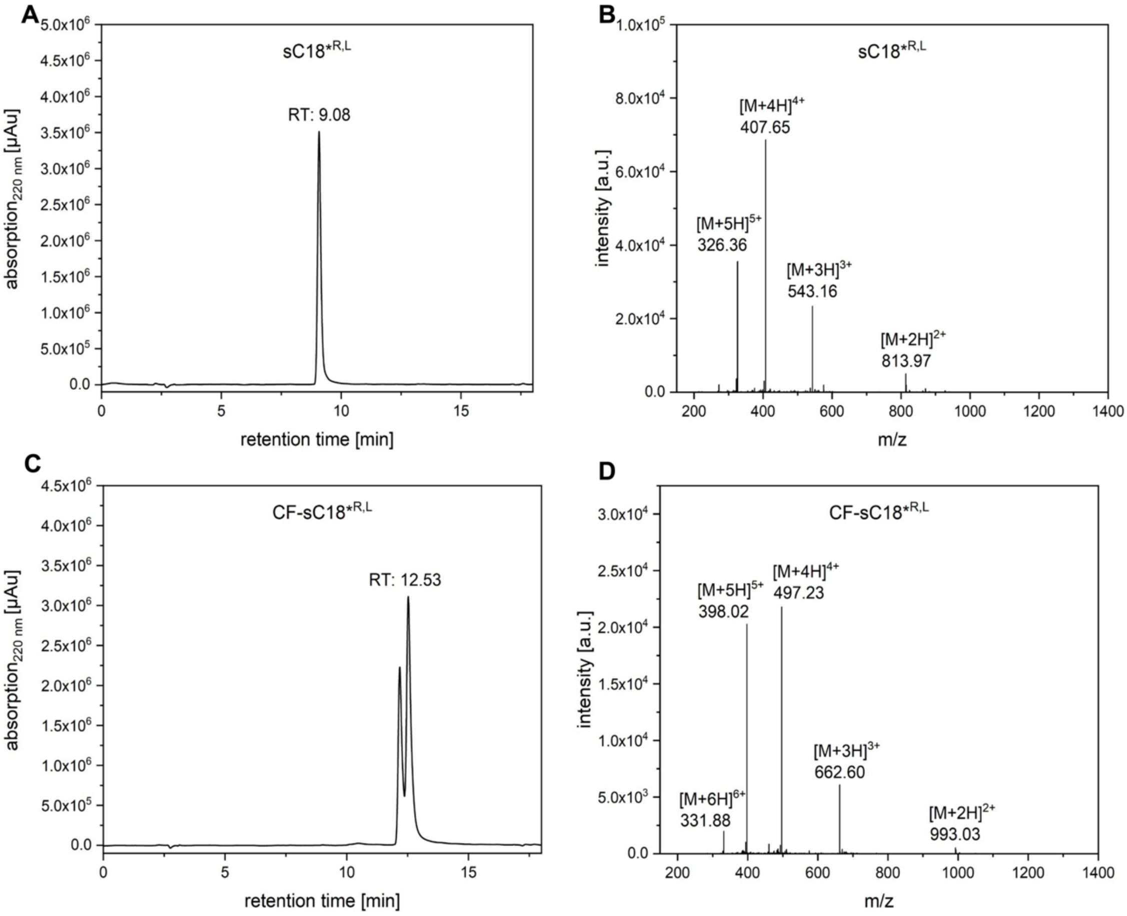

Figure S8 LC-MS analysis showing UV chromatographs was recorded using a linear gradient from 10–60% ACN in water (incl. 0.1% TFA) over 15 min (A, B). Mass spectra of sC18^{*R,L} and CF-sC18^{*R,L} (C, D), respectively.

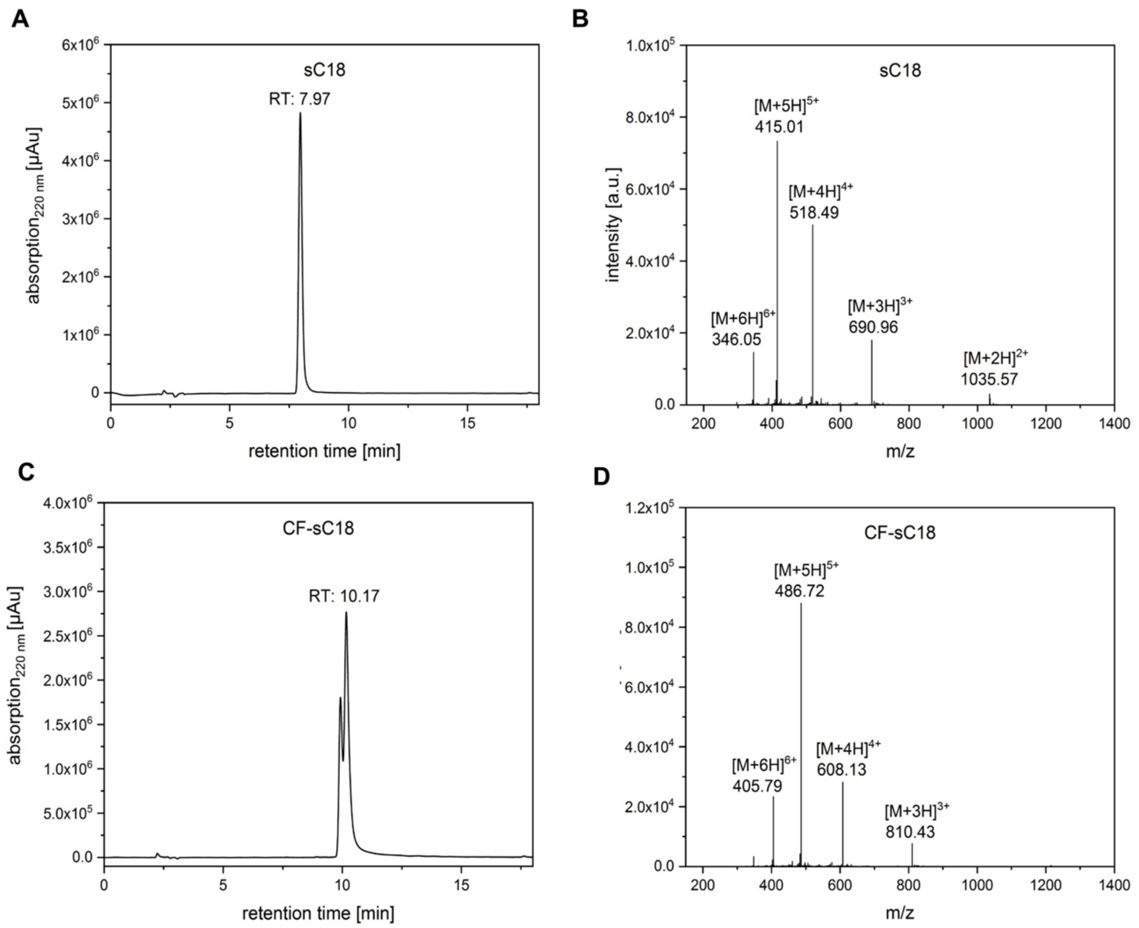

Figure S9 LC-MS analysis showing UV chromatographs was recorded using a linear gradient from 10–60% ACN in water (incl. 0.1% TFA) over 15 min (A, B). Mass spectra of sC18 and CF-sC18 (C, D), respectively.

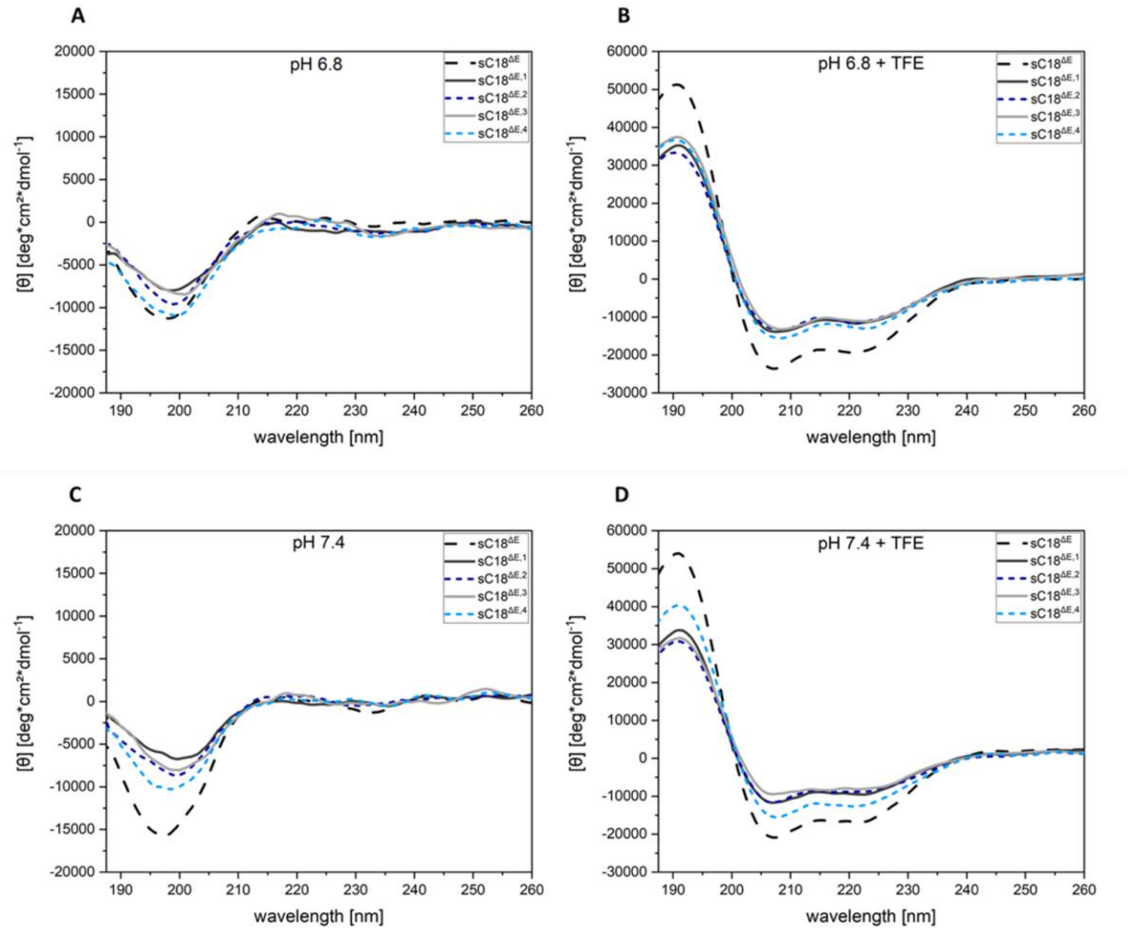

Figure S10 CD spectra of sC18$^{\Delta E}$ and all histidine variants in different media (potassium phosphate buffer: A, C and phosphate buffer with the addition of 50 % TFE: B, D) and at different pH values (pH 6.8: A, B; pH 7.4: C, D).

Table S1 Calculated R-values [$\frac{[\theta]_{220\ nm}}{[\theta]_{207\ nm}}$] of the synthesized peptides in potassium phosphate buffer with 50 % TFE at pH 6.8 and 7.4.

	**R-value**	
**Peptide**	**pH 6.8 +TFE**	**pH 7.4 +TFE**
**sC18$^{\Delta E}$**	0.81	0.81
**sC18$^{\Delta E,1}$**	0.84	0.82
**sC18$^{\Delta E,2}$**	0.88	0.76
**sC18$^{\Delta E,3}$**	0.87	0.86
**sC18$^{\Delta E,4}$**	0.85	0.81

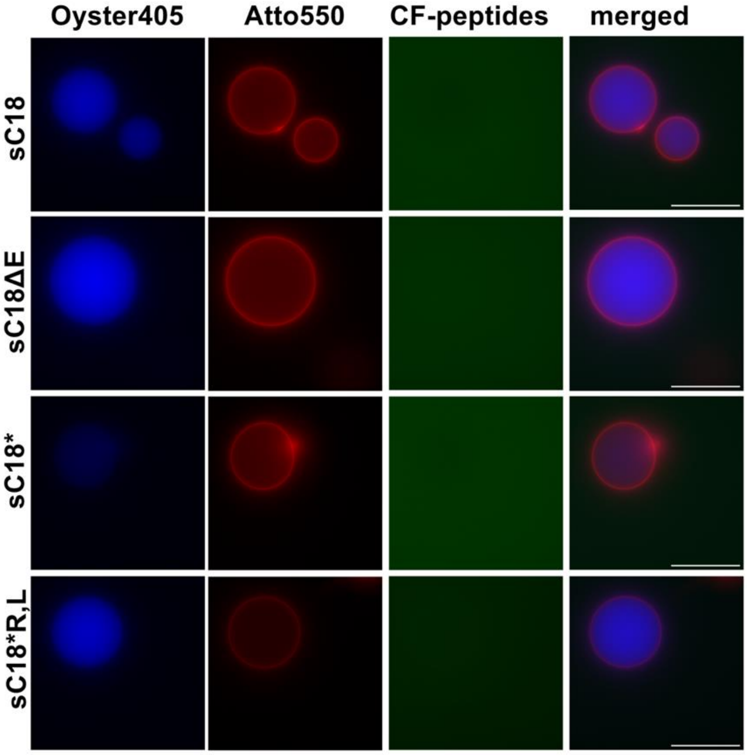

Figure S11 Peptide interaction with giant lamellar vesicles (GUVs) composed of DOPC/DOPE (50:50). 1 µM solutions of sC18, sC18$^{\Delta E}$, sC18* and sC18*^{R,L} were incubated with GUVs for 30 min and inspected using a fluorescence microscope (Keyence). Red: Atto550; green: CF-labeled peptides, blue: Oyster 405. Scale bar: 50 µm.

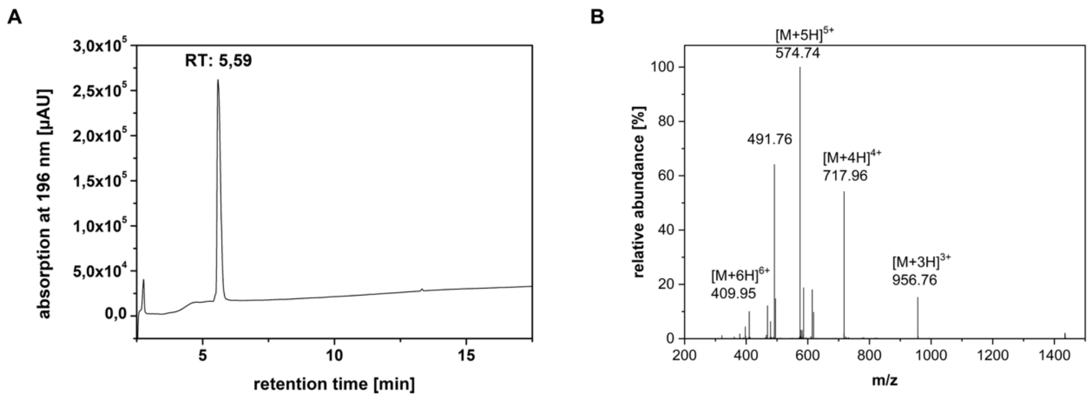

Figure S12 LC-MS analysis showing UV chromatograph (A) and corresponding mass spectra (B) of PDC-1. UV-chromatograph was recorded using a linear gradient from 10–60% ACN in water (incl. 0.1% FA) over 15 min.

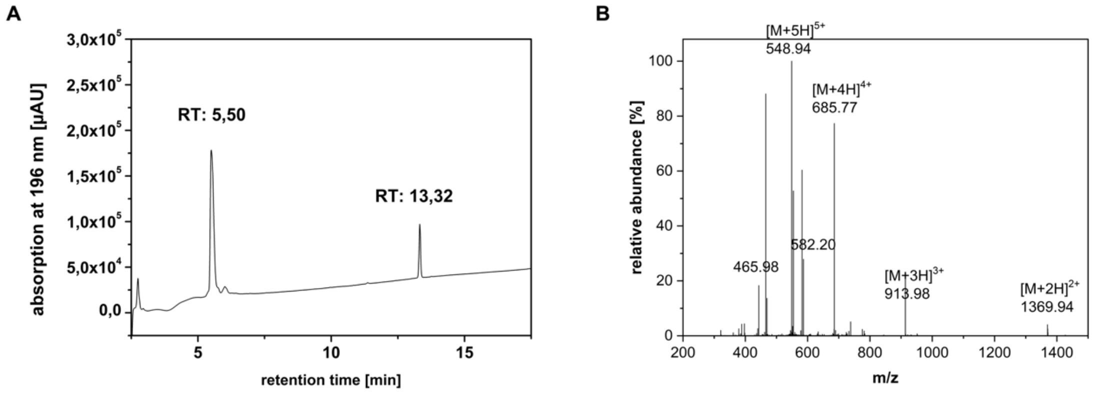

Figure S13 LC-MS analysis showing UV chromatograph (A) and corresponding mass spectra (B) of PDC-2. UV-chromatograph was recorded using a linear gradient from 10–60% ACN in water (incl. 0.1% FA) over 15 min.

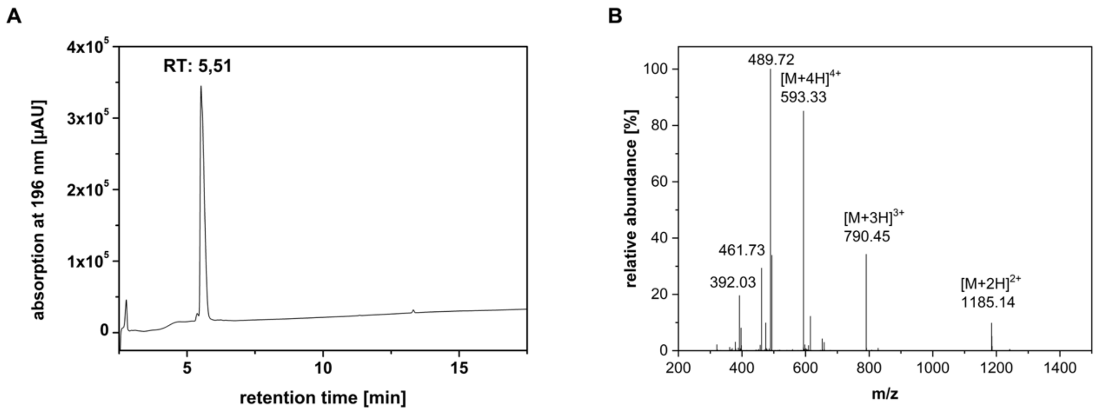

Figure S14 LC-MS analysis showing UV chromatograph (A) and corresponding mass spectra (B) of PDC-3. UV-chromatograph was recorded using a linear gradient from 10–60% ACN in water (incl. 0.1% FA) over 15 min.

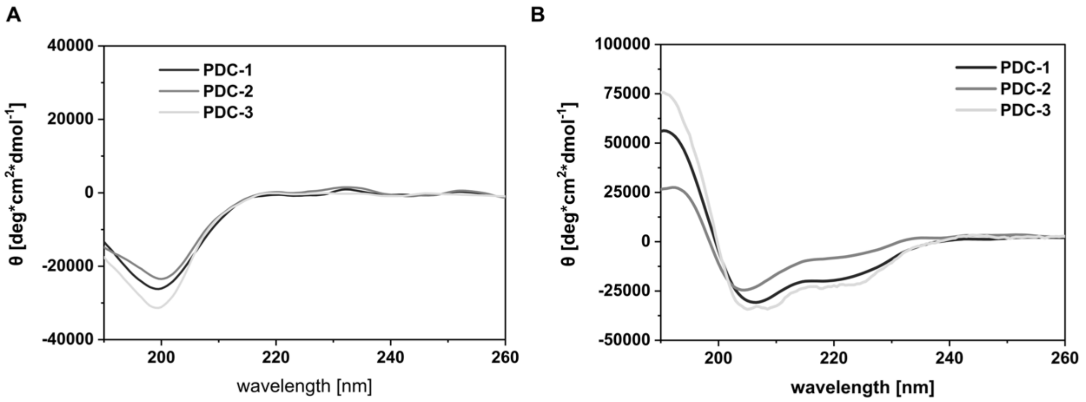

Figure S15 CD spectra of all PDCs in different media (potassium phosphate buffer (pH: 7): A and potassium phosphate buffer (pH: 7) with the addition of 50 % TFE: B)
